# Colorimetric LAMP microfluidic chip for detecting three allergens: peanut, sesame and soybean

**DOI:** 10.1038/s41598-018-26982-5

**Published:** 2018-06-06

**Authors:** Dan Yuan, Jilie Kong, Xinxin Li, Xueen Fang, Qin Chen

**Affiliations:** 1 Shanghai Key Laboratory of Bio-Energy Crops, School of Life Sciences, Shanghai University, Shanghai, 200444 P.R. China; 2 Department of Chemistry and Institutes of Biomedical Sciences, Fudan University, Shanghai, 200433 P.R. China; 3 Shanghai Suchuang Diagnostics Co., Ltd., Shanghai, 201318 P.R. China

## Abstract

Food allergies can greatly harm people’s health, and therefore detecting allergens in foods is extremely important. By integrating loop-mediated isothermal amplification (LAMP) with a microfluidic chip, we have developed a method for detecting the allergen genes of peanut (*Arachis hypogaea*), sesame (*Sesamum indicum*), and soybean (*Glycine max*) using a colorimetric method suitable for the naked eye, known as the colorimetric LAMP microfluidic chip. In the presence of peanut, sesame, or soybean in the samples, the corresponding reaction well of the microfluidic chip will appear pink, or otherwise remain light brown. This method of detection is specific and can easily distinguish these three allergens from others in foods. The detection limit for peanut, sesame and soybean allergens was 0.4 ng/μL using the LAMP-microfluidic chip. The accuracy of this novel and rapid method was validated using allergenic foods obtained commercially and was comparable with that of the typical TaqMan real-time PCR method.

## Introduction

Food allergy is an abnormal immune response to certain foods, where the corresponding immunoglobulin binding to the food releases several chemicals that cause allergenic symptoms such as nausea, vomiting, and eczema. Allergens commonly exist in foods such as milk, eggs, peanuts, soybeans, walnuts, almonds, hazelnuts, sesame seeds, and wheat^1,2^. Among these, the allergenic proteins of peanuts are mainly Ara h 1, Ara h 2, Ara h 3, and Ara h 6^3^; that of sesame is 2S albumin^4^, while those of soybean include Gly m Bd 28 K, Gly m Bd 30 K, and β-globulin Gly m Bd 60 K^5^. These allergens in food can easily cause health problems for allergic people^6^, and therefore developing accurate methods for their detection is essential. Common methods for detecting allergenic protein components are enzyme-linked immunosorbent assay (ELISA)^7^, matrix-assisted laser desorption ionization – time-of-flight/mass spectrometry (MALDI-TOF/MS)^8^, and western blotting^9^, while other techniques can detect the allergen gene, for example, polymerase chain reaction (PCR)^10^, genetic maker^11–13^, gene microarray chip^14^ and pyrosequencing^15^. All these methods can be cumbersome to use and require expensive equipment. Isothermal nucleic acid detection includes methods such as strand displacement amplification (SDA)^16^, recombinase polymerase amplification (RPA)^17^, nucleic acid sequence-based amplification (NASBA)^18^, rolling circle amplification (RCA)^19^, and loop-mediated isothermal amplification (LAMP)^20^. In recent years, LAMP, a nucleic acid detection technology, has been established by Notomi *et al*. (2000) for detecting and identifying viruses, bacteria, animals and plants^21–23^. Under *Bst* DNA polymerase, they designed four specific primers to trigger the isothermal amplification (60–65 °C) of the target DNA within 60 min. This amplification efficiency is significantly greater than that of traditional PCR techniques.

Microfluidic chip technology has also rapidly developed in recent years^24,25^. Initially, the microfluidic chip had individual functional modules, for example for nucleic acid purification, its amplification, then target detection. At present, research has focused on integrating each functional module into a single device^26,27^. For example, centrifugal force- and capillary force-driven microfluidic chips have been designed to enable real-time and easy detection to be combined with LAMP technology^28^. The only equipment required is a simple centrifuge and a thermostatically-controlled water bath (or oven); no other expensive equipment is needed^29^.

In the present study, we aim to combine LAMP with microfluidic chip technology^30^, to develop a colorimetric LAMP microfluidic chip for simultaneously detecting peanut, sesame and soybean allergens in one chip^31^. This technology will be tested using allergenic foods commercially available to consumers.

## Results and Discussion

### Screening primers for three allergens of peanut, sesame and soybean

We screened a total of 12 sets of LAMP primers for the different allergens, selecting one for each allergen: EF609643.1, EU493458.1, and AF240005.1 from the NCBI GenBank database for Ara h 6 (peanut), allergen 2 S albumin (sesame), and Gly m Bd 28 K (soybean), respectively (Table 1) to provide the following results.

**Table 1:** Sequences for LAMP primers used for detecting peanut, sesame and soybean allergens.

Primer name	Primer sequence	
Peanut (Ara h 6)	F3	ATCTTCATTGATCATATAGCACA
	B3	GGCTTAGTATGTGAGGTACG
	FIP	ATGCAAATACTCCAAGATTCCCATT-TAATTACTACAGCAAAGCCTGA
	BIP	GCATGAAAATGTAACGTGGAAGC-TAAAGGGAATGGAGGGTGG
Sesame (2 S albumin)	F3	GGAACGTGGACGAGAGGT
	B3	CGCTTGGTTGATTGCGATTC
	FIP	GATTGGCCCTCCTGGTAGCC-GCTGTGAGGCCATTAGGC
	BIP	ACAGCAGGTTTACCAGAGGGC-ATTGGCATTGCTGGGGTC
Soybean (Gly m Bd 28 K)	F3	GGAAGCAAAGCTGGGATT
	B3	GAAATATGTGAACTTATGATGGATG
	FIP	TGAACCAGATGGAATCATGTACAA-TGATGATGAACTAGCGGAA
	BIP	TAGGAGAAGGTCAGAGACTTCACG-ATGCATGTACCTGGAAGG

**Figure 1:**
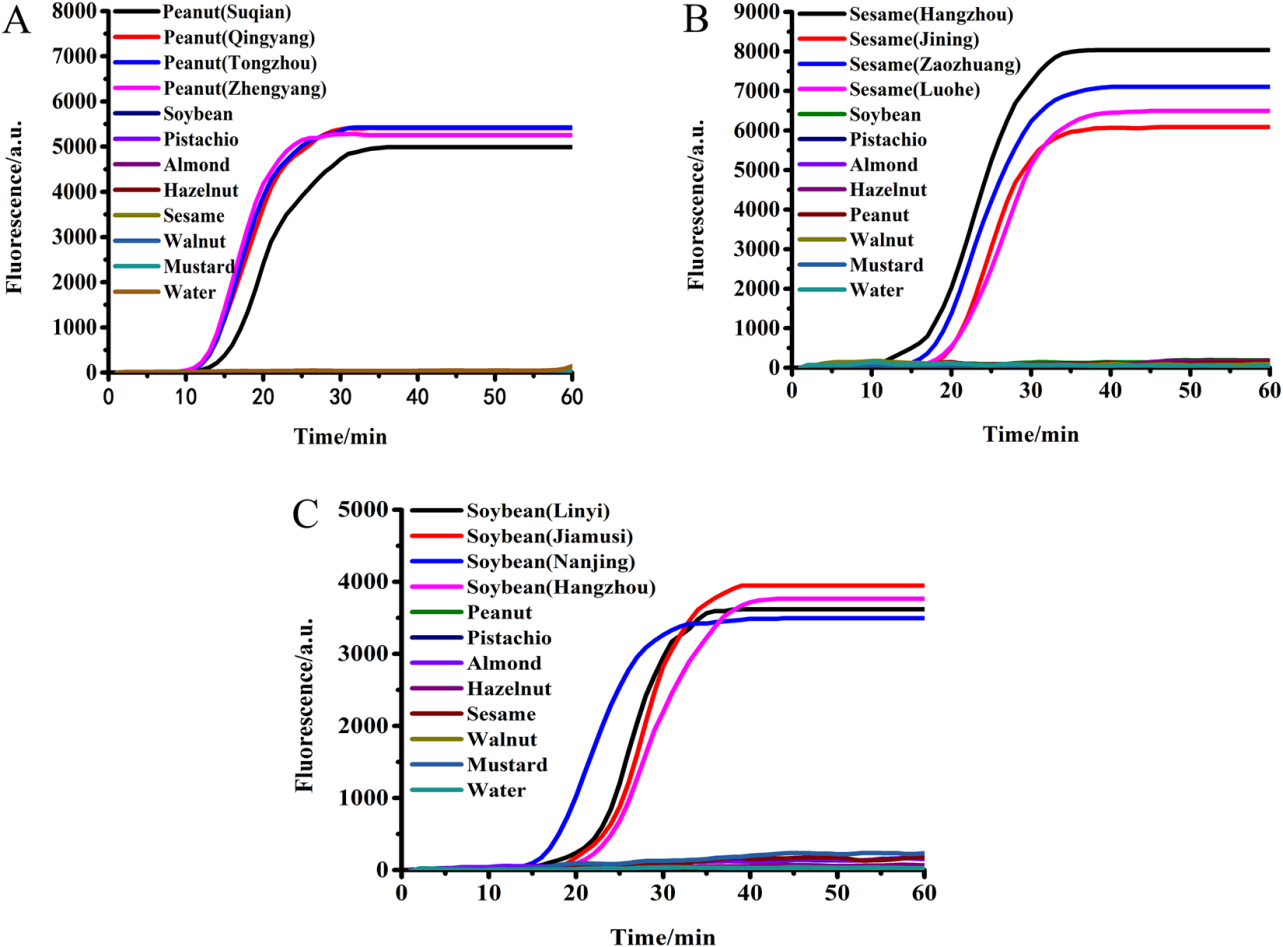
Results of screening for primers of (**A**) peanut, (**B**) sesame, and (**C**) soybean purchased from different locations. 10 ng/μL, 5 μL of tested samples were added in each reaction.

After the reaction at 63 °C, the fluorescence signals were collected. Figure 1A,B,C indicated the test results for peanut, sesame, and soybean, respectively. It was clear that the results from peanut, sesame and soybean purchased from different locations could be amplified, which were detected at about 10 min, 15 min, and 20 min, respectively, then reaching a peak at around 25 min, 30 min and 35 min, respectively. No amplification was detected in the negative controls. This showed that the primers were designed for the peanut, sesame and soybean allergen genes and therefore could be used for detecting these three allergens.

### Specificity of the colorimetric LAMP-microfluidic chip for three allergens of peanut, sesame and soybean

**Figure 2:**
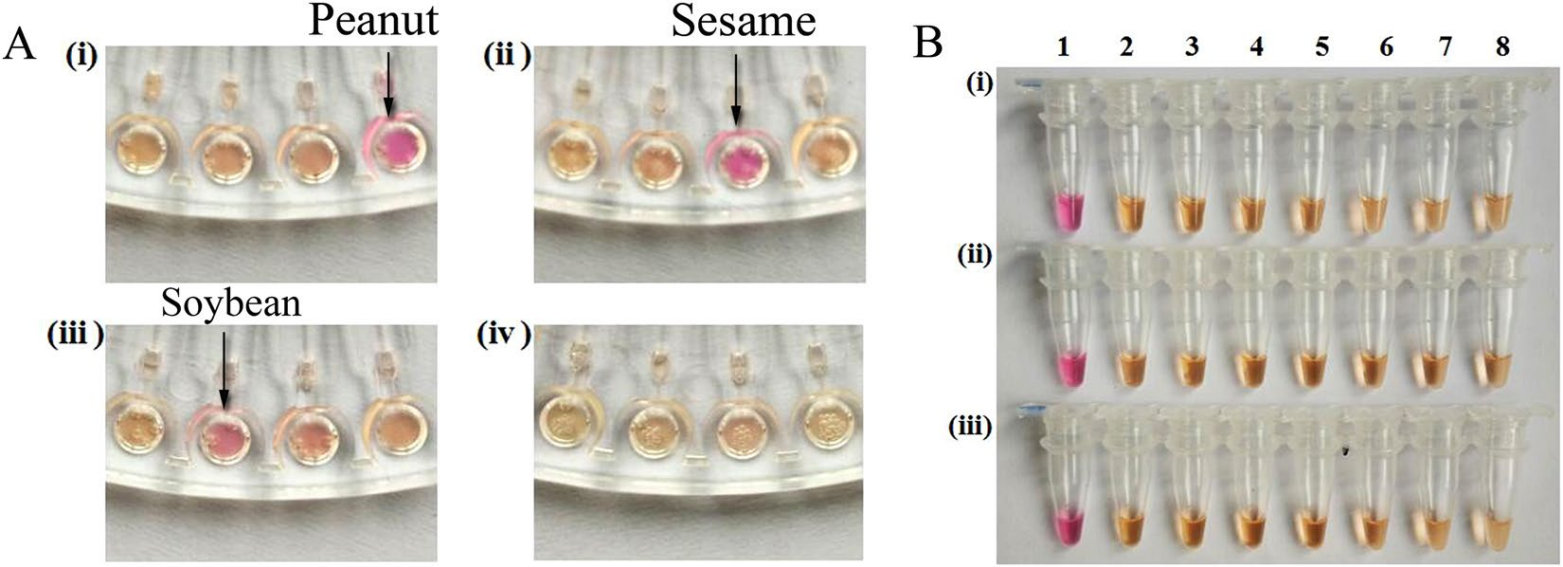
Results of the colorimetric test for the specificity of the LAMP-microfluidic chip detection method. 10 ng/μL, 5 μL of tested samples were added in each reaction. (**A**) (i)–(iv) indicate the results for peanut, sesame, soybean and blank control, respectively. The primers from right to left were for peanut, sesame, and soybean allergen and control water, respectively. In (i)–(iv) the DNA of peanut, sesame, soybean and control water were added, respectively. (**B**) (i), (ii) and (iii) indicate that the added primers were for the peanut, sesame and soybean allergen genes, respectively. For columns 1-8 in row (i), the tested samples were peanut, pistachio, soybean, almond, hazelnut, sesame, walnut, and mustard, respectively; in row (ii), sesame, peanut, pistachio, soybean, almond, hazelnut, walnut, and mustard, respectively; and in row (iii), soybean, peanut, pistachio, almond, hazelnut, sesame, walnut, and mustard, respectively.

After designing and screening the primers for peanut, soybean and sesame allergen genes, a set of LAMP primers for the three allergens was obtained which could accurately distinguish between the positive and negative samples. On this basis, the LAMP amplification system was combined with the microfluidic chip for use with the naked eye. NeuRed dye, a kind of pH indicator, which exhibits a red color when the pH of the reaction buffer is less than 8.0^32^, was used to indicate a result by a color change. The pH of the initial reaction buffer was 8.8. When the LAMP reaction occurred, a lot of hydrogen ions were produced and the solution gradually became acidic to appear pink; otherwise it maintained its original light brown color. The primers for peanut, sesame, and soybean and the control water were put in the wells from right to left then the DNA of peanut, sesame, soybean and control water was transported into the sample well of the microfluidic chip. Figure 2A shows that a pink color appeared in (i), (ii), and (iii), indicating that peanut, sesame and soybean allergens had been detected, respectively^33^, while the light brown color indicated a negative reaction. To check the feasibility of the LAMP-microfluidic chip for detecting the three allergens of peanut, sesame and soybean, we performed LAMP colorimetric detection using typical PCR tubes. The results shown in Fig. 2B were consistent with the microfluidic-LAMP assay, with the positive samples detected for peanut, sesame, and soybean allergens. This indicated that, for the LAMP-microfluidic chip detection method, it was extremely easy to observe the test results without needing expensive equipment.

### Sensitivity of colorimetric LAMP-microfluidic chip for three allergens of peanut, sesame and soybean

**Figure 3:**
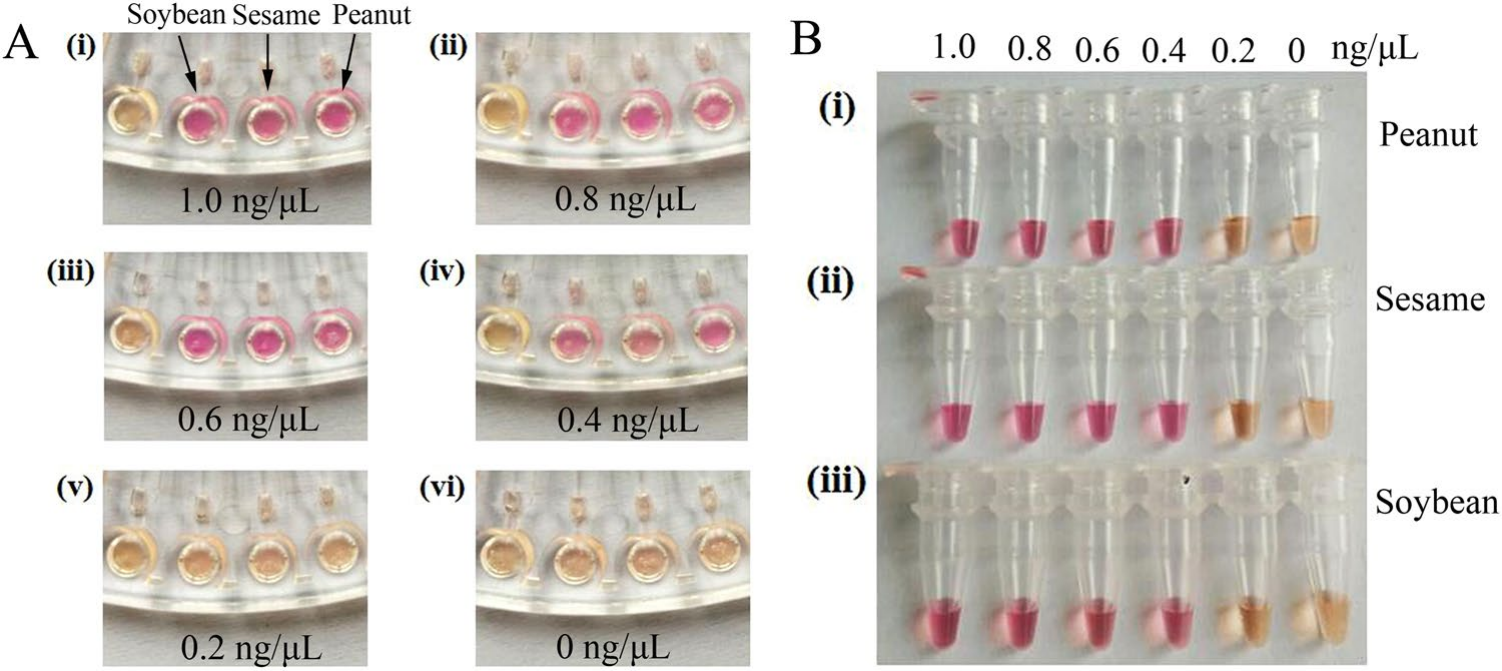
Results of the colorimetric test for the sensitivity of the LAMP-microfluidic chip detection method. (**A**) Sensitivity for mixed DNA samples of peanut, sesame and soybean in LAMP-microfluidic chips. From right to left, allergen primers of peanut, sesame, soybean and control water were added to each. The concentration gradient of the mixed DNA sample was 1, 0.8, 0.6, 0.4, 0.2, and 0 ng/μL from (i) to (vi), respectively. (**B**) For (i) to (iii) the primers added were the allergen primers for peanut, sesame and soybean, respectively. The concentration gradient of the mixed DNA sample was 1, 0.8, 0.6, 0.4, 0.2, and 0 ng/μL.

After evaluating the specificity of the colorimetric data from the microfluidic-LAMP chip detection method, we investigated its sensitivity. The results in Fig. 3A show that the sensitivity of the LAMP-microfluidic chip for the mixed DNA samples of peanut, sesame and soybean allergens was 0.4 ng/μL. It should be noted that the concentration of the samples tested from (i) to (iv) changed in a descending gradient, and correspondingly, the color of the test results gradually became somewhat lighter, but not obviously so. From this, the sensitivity of detection of peanut, sesame and soybean in the tubes was 0.4 ng/μL, a result proving the feasibility of the LAMP-microfluidic chip detection method (Fig. 3B). As a comparison, the sensitivity of the real-time PCR method was 0.01 ng/μL (Supplementary Information Fig. S1), but the on-chip colorimetric LAMP method could distinguish the results by naked-eye, which did not need an expensive device. So the method is very convenient.

### Application of the LAMP microfluidic chip detection method to real foods

**Figure 4:**
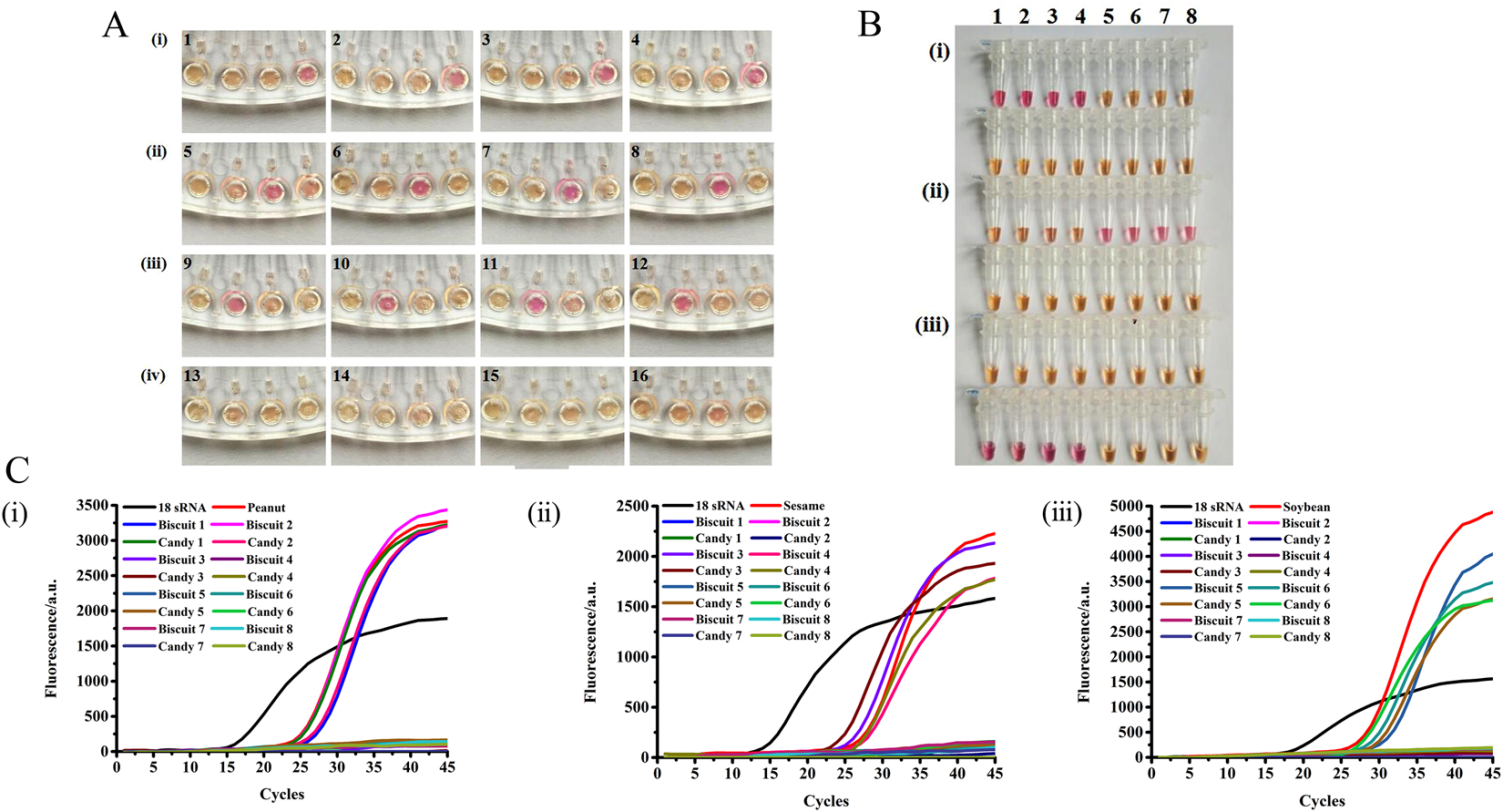
Results for detecting allergens in commercial food products using three different methods. (**A**) LAMP microfluidic chip detection. From right to left, allergen primers of peanut, sesame, soybean and control water were added to each well. 1-16 were biscuit 1, biscuit 2, candy 1, candy 2, biscuit 3, biscuit 4, candy 3, candy 4, biscuit 5, biscuit 6, candy 5, candy 6, biscuit 7, biscuit 8, candy 7, and candy 8, respectively. (**B**) LAMP in tubes. Primers detecting peanut, sesame, and soybean allergens were added for (i)–(iii) with 1-16 as for A. (**C**) Test results using traditional RT-PCR with primers for detecting peanut, sesame, and soybean allergens added for (i)–(iii).

The specificity and sensitivity test results have illustrated that the LAMP-microfluidic chip detection system was stable, rapid, convenient and feasible. To verify the actual performance of the system, we tested it using sixteen different kinds of allergenic food bought from a supermarket: eight kinds of biscuit and eight kinds of candy. After extracting their DNA and mixing it with the LAMP test solution, we tested for allergens using our method. The color change in the reaction wells of the chip could be seen after incubation in a water bath for 60 min. Figure 4A shows that peanut was detected in biscuit 1, biscuit 2, candy 1, and candy 2; sesame in biscuit 3, biscuit 4, candy 3, and candy 4; and soybean in biscuit 5, biscuit 6, candy 5, and candy 6. The remaining four kinds of food contained none of these three allergens. At the same time, we also conducted the LAMP amplification experiments in tubes. Figure 4B shows that the 16 samples in B (i), (ii) and (iii) had four kinds of products containing peanut, sesame and soybean allergens. To further confirm the feasibility of the LAMP-microfluidic chip method, we also performed traditional RT-PCR (Fig. 4C). In the 16 samples, the RT-PCR method also detected that each had four kinds of food containing peanut, sesame and soybean allergens, results which were consistent with those of the LAMP-microfluidic chip assay (Table 2).

**Table 2:** Comparison of three methods for detecting allergens of peanut, sesame, and soybean in a range of commercial foods.

sample	LAMP-microfluidic chip	Typical-LAMP assay	traditional RT-PCR	sample	LAMP-microfluidic chip	Typical-LAMP assay	traditional RT-PCR
Biscuit 1	+−−	+−−	+−−	Candy 1	+−−	+−−	+−−
Biscuit 2	+−−	+−−	+−−	Candy 2	+−−	+−−	+−−
Biscuit 3	−+−	−+−	−+−	Candy 3	−+−	−+−	−+−
Biscuit 4	−+−	−+−	−+−	Candy 4	−+−	−+−	−+−
Biscuit 5	−−+	−−+	−−+	Candy 5	−−+	−−+	−−+
Biscuit 6	−−+	−−+	−−+	Candy 6	−−+	−−+	−−+
Biscuit 7	−−−	−−−	−−−	Candy 7	−−−	−−−	−−−
Biscuit 8	−−−	−−−	−−−	Candy 8	−−−	−−−	−−−

Note: “+”, positive; “−”, negative. Each cell in the table indicates three results for the presence or absence of peanut, sesame and soybean. For example, “+ − −” shows that the sample contained peanut allergen, but no allergens for sesame and soybean.

## Conclusions

Overall, a microfluidic-LAMP test^34,35^ has been successfully established for the rapid and accurate detection of three allergens of peanut, soybean and sesame with a high specificity and sensitivity within 60 min. This method was also applied to test for the presence or absence of allergens in foods purchased on the open market. Its accuracy was comparable to the traditional TaqMan real-time PCR method. The establishment of this new method will help to improve the effective supervision of food allergens by food inspection departments. This will help consumers to avoid buying and eating foods containing allergenic substances, thus leading to a lower risk of causing them injury and illness.

## Methods

### Samples and reagents

Three kinds of foods resulting in allergic reactions were tested: peanuts (purchased from Suqian City, Jiangsu Province; Qingyang City, Gansu Province; Tongzhou District, Beijing; and Zhengyang City, Henan Province, China), sesame seeds (purchased from Hangzhou City, Zhejiang Province; Jining City, Shandong Province; Zaozhuang City, Shandong Province; and Luohe City, Henan Province, China), and soybeans (purchased from Linyi City, Shandong Province; Jiamusi City, Heilongjiang Province; Nanjing City, Jiangsu Province; and Hangzhou City, Zhejiang Province, China). Pistachios (*Pistacia vera*), almonds (*Amygdalus Communis Vas*), hazelnuts (*Corylus heterophylla Fisch*), walnuts (*Juglans regia*), mustard seeds (*Brassica juncea*), eight kinds of biscuit, and eight kinds of candy were also purchased from local supermarkets. The LAMP working buffer (20 mM Tris-HCl (pH 8.8, 25 °C), 10 mM KCl, 10 mM (NH_4_)_2_SO_4_, 8 mM MgSO_4_, 0.1% Tween-20), SYBR Green I, NeuRed dye (500 μM) were obtained from Shanghai Suchuang Diagnostics Co. Ltd. (Shanghai, China), *Bst* DNA polymerase (8000 units) from New England Biolabs (Shanghai, China) and the RT-PCR kit from Sangon Biotech (Shanghai) Co. Ltd. (Shanghai, China).

### DNA extraction and sample preparation

One hundred and fifty milligrams of the test material were placed in mortar then, after adding a small amount of liquid nitrogen, were quickly ground to a powder. The powder was then placed in a 1.5-mL centrifuge tube with 600 μL of CTAB extracting solution (100 mM Tris-HCl, pH 8.0, 20 mM Na_2_EDTA, 1.4 M NaCl, 20 g/L CTAB)^36^, and 15 μL of proteinase K (20 mg/mL) then incubated at 65 °C for 30 min. An equal volume of a mixture of phenol–chloroform–isoamyl alcohol (25:24:1, v/v) was added. After vigorous shaking, the mixture was centrifuged at 12000 *g* for 15 min. The supernatant was then transferred to a fresh tube, and an equal volume of chloroform: isoamyl alcohol (volume ratio of 24:1) was added. After vigorous shaking, it was centrifuged again at 12000 *g* for 15 min. After removing the supernatant to a new tube, an equal volume of isopropanol was added to precipitate the DNA with centrifuging at 12000 *g* for 10 min. The supernatant was then removed and the precipitate washed twice with cold 70% ethanol, dried at ambient temperature, and dissolved in about 100 μL TE buffer. The DNA concentrations were determined spectrophotometrically using a NanoDrop 1000 spectrophotometer (Thermo Scientific, Waltham, MA, USA). The DNA sample was diluted to 10 ng/μL then stored at −20 °C.

### Primer design and reaction system establishment

The gene numbers of the Ara h 6 (peanut), allergen 2 S albumin (sesame), and Gly m Bd 28 K (soybean) were EF609643.1, EU493458.1 and AF240005.1 (taken from the NCBI GenBank database). Using these gene sequences, four specific primers were designed by the online software PrimerExplorer V5 (primerexplorer.jp/e/) (Table 1). All primers were synthesized by Thermo Fisher Scientific (China) Co. Ltd (Beijing, China). The concentration of the four primers was 100 μM. FIP/BIP: F3/B3: water were mixed in the ratio 8:4:1 then stored at −20 °C. For a comparison with the LAMP detection method, we also performed traditional RT-PCR. The primers included forward and reverse primers and the fluorescent probes of 18 rRNA, peanut, sesame and soybean to comply with the entry and exit inspection and quarantine industry standards of the People’s Republic of China.

### Microfluidic chip design

**Figure 5:**
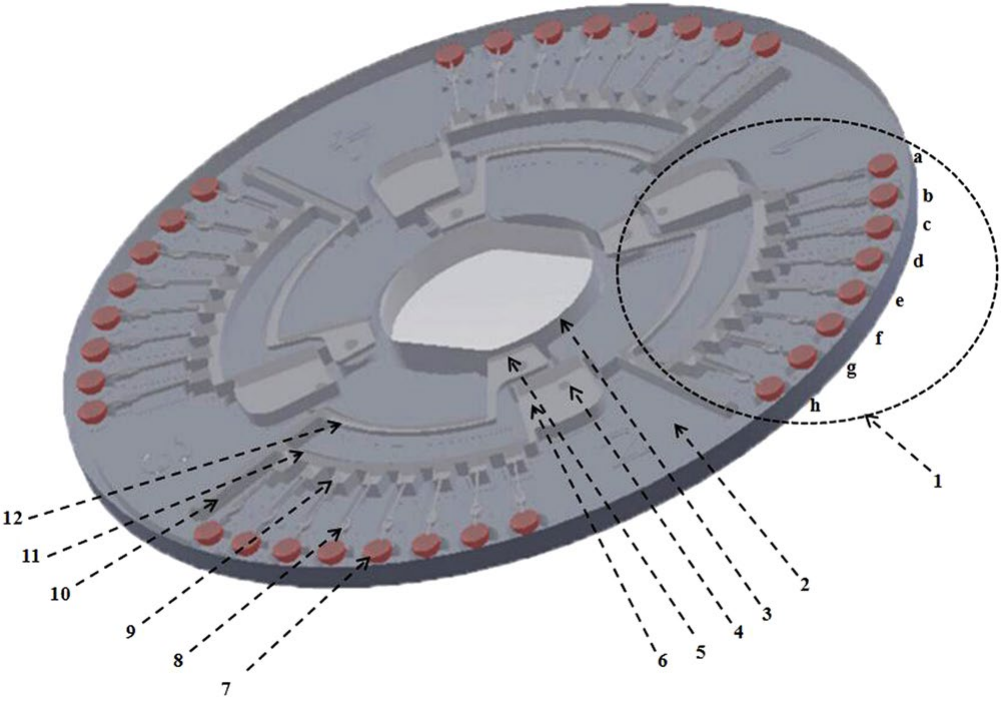
Structure of the microfluidic chip (1: reaction detection section, 2: negative film, 3: through hole, 4: sample well, 5: vent hole, 6: storage zone, 7: reaction well, 8: ball valve, 9: reserved zone, 10: waste liquid well, 11: second arc-shaped channel, 12: first arc-shaped channel).

The material of the microfluidic chip is polymethyl methacrylate (PMMA). The diameter of the microfluidic chip is 80 mm. The microfluidic chip (Fig. 5) is disc-shaped, with four reaction detection sections, each of which comprises a sample well, a vent, and eight reaction wells (a-h). The diameter of each reaction well is 3.0 mm. The volume of each reaction well is 5 μL. To be compatible with the microfluidic chip, the rotor of the centrifuge device is transformed from ordinary centrifuge device. The chip is centrifuged through different injection wells. The reserved zone is connected to the reservoir through the ball valve then centrifuged at a low speed (1000 *g*). The liquid in the storage zone flows into the reserved zone because of the centrifugal force. The reserved zone communicates with the reaction well through the ball valve and is centrifuged at high speed (3000 *g*). The liquid in the reserve zone flows into the reaction well because of the centrifugal force, and the excess liquid flows into the waste liquid well. When the reaction is performed at a certain temperature and the heating enters the reaction stage, the ball valve prevents the liquid in the sample well from flowing back into the reserved zone and then diffusing into the surrounding reaction hole while still ensuring that the liquid in the reaction well is full. In the present study, the reactions were carried out normally and contamination was prevented.

### Primer screening and establishment of LAMP system

Twenty-five microliters of the reaction mixture was prepared, comprising 17.7 μL of the LAMP working buffer (containing fluorescent dye SYBR Green I), 1.3 μL of primer solution, 1 μL of *Bst* DNA polymerase and 5 μL of the sample being tested. Three groups of 12 detection tests were performed using the same primers of peanut, sesame, and soybean. The different samples used the following settings. (1) Four positive controls were set up with peanut template DNA, and pistachio, soybean, hazelnut, almond, sesame, walnut, mustard, and water were used as the negative controls. (2) Four positive controls were set up with soybean template DNA, and pistachio, peanut, hazelnut, almond, sesame, walnut, mustard and water were used as the negative controls. (3) The detection of sesame also used similar methods to set up the control tests. The prepared system was homogenized, centrifuged so that no bubbles were present in the tubes then reacted at 60–65 °C for 60 min. The fluorescence signal was collected using a fluorescence quantitative PCR instrument (LineGene 9640, Bioer, Hangzhou, China), maintained at 95 °C for 5 min then finally falling to room temperature at 25 °C.

### Detection of allergens using the LAMP-microfluidic chip

The primers for the peanut, sesame and soybean allergen genes were deposited in the microfluidic chip. The eight reaction wells, a-h (Fig. 5), contained the primers of peanut, sesame, soybean, control water, peanut, sesame, soybean and control water, respectively. The other three reaction detection sections were labeled in the same order. Seventy-five microliters of the reaction system of the sample was tested (3 times the volume of the mixed system, where the primer liquid was replaced with water). This was mixed then added to the sample wells of the chip, and a negative control was set. After centrifuging the mixture into the reaction wells, the excess mixture automatically flowed into the waste liquid well. The holes of the chip were sealed with adhesive tape then the chip was incubated in a 63 °C water bath (or oven) for 60 min. A well turning pink indicated the presence of a corresponding allergen in the sample. All the wells, a-d or e-h on the chip, would be examined for the experimental results.

### Specificity of colorimetric detection

Peanuts, soybeans, sesame seeds, pistachios, almonds, hazelnuts, walnuts, and mustard seeds were tested to evaluate the specificity of the colorimetric detection for this method. A total of 25 μL of the reaction mixture was prepared as follows: 17.7 μL of the LAMP reaction buffer (containing 1 μL of NeuRed dye), 1.3 μL of the primer solution, 1 μL of the *Bst* DNA polymerase, and 5 μL of the DNA sample. Four sets (peanut, sesame, soybean and water) were set up for testing, with 75 μL (3 times the volume of the above system) of the mixed reaction liquid being added to each of the reaction wells of the chip. Three groups of tests were conducted in tubes, using the same peanut, sesame and soybean primers for each group. Eight samples were tested with 25 μL of the reaction mixture being added to each. The color change after the end of reaction will appear pink for a positive sample and light brown for a negative sample.

### Sensitivity of colorimetric detection

For peanut, sesame and soybean, a series of concentrations of 1, 0.8, 0.6, 0.4, 0.2, and 0 ng/μL was prepared, to use for evaluating the sensitivity of a typical-LAMP assay, the microfluidic-LAMP assay and a typical RT-PCR assay.

### Traditional RT-PCR

The traditional RT-PCR assay^37,38^ was used to compare and evaluate the LAMP-microfluidic test using allergenic foods purchased on the open market. The detection procedure was carried out according to the RT-PCR protocols (as set out in standards SN/T 1961.2-2007, SN/T 1961.12-2013, SN/T 1961.19-2013, People ‘s Republic of China Entry-Exit Inspection and Quarantine Industry Standard). The primers and probes were reported in Supplementary Information Table S1.

## Electronic supplementary material

Supplementary Information
