# Supplementary material for: Colorimetric LAMP microfluidic chip for detecting three allergens: peanut, sesame and soybean
Source: Sci Rep. 2018 Jun 6;8:8682. doi: 10.1038/s41598-018-26982-5 (PMC5989197; doi:10.1038/s41598-018-26982-5)
Supplement: Supplementary file 1 — Supplementary Information [file 41598_2018_26982_MOESM1_ESM.docx]

**Colorimetric LAMP microfluidic chip for detecting** **three allergens: peanut, sesame and soybean**

Dan Yuan^1^, Jilie Kong^2^, Xinxin Li^3^, Xueen Fang^2^*, Qin Chen^1^*

*^1^Shanghai Key Laboratory of Bio-Energy Crops, School of Life Sciences, Shanghai University, Shanghai, P.R. China*

*^2^Department of Chemistry and Institutes of Biomedical Sciences, Fudan University, Shanghai 200433, P.R. China*

*^3^Shanghai Suchuang Diagnostics Co., Ltd., Shanghai 201318, P.R. China*

* Correspondence should be addressed to Q.C [([chenqincc@staff.shu.edu.cn](mailto:chenqincc@staff.shu.edu.cn))](mailto:(jlkong@fudan.edu.cn)) or X.E.F. [(fxech@fudan.edu.cn)](mailto:(fxech@fudan.edu.cn)).


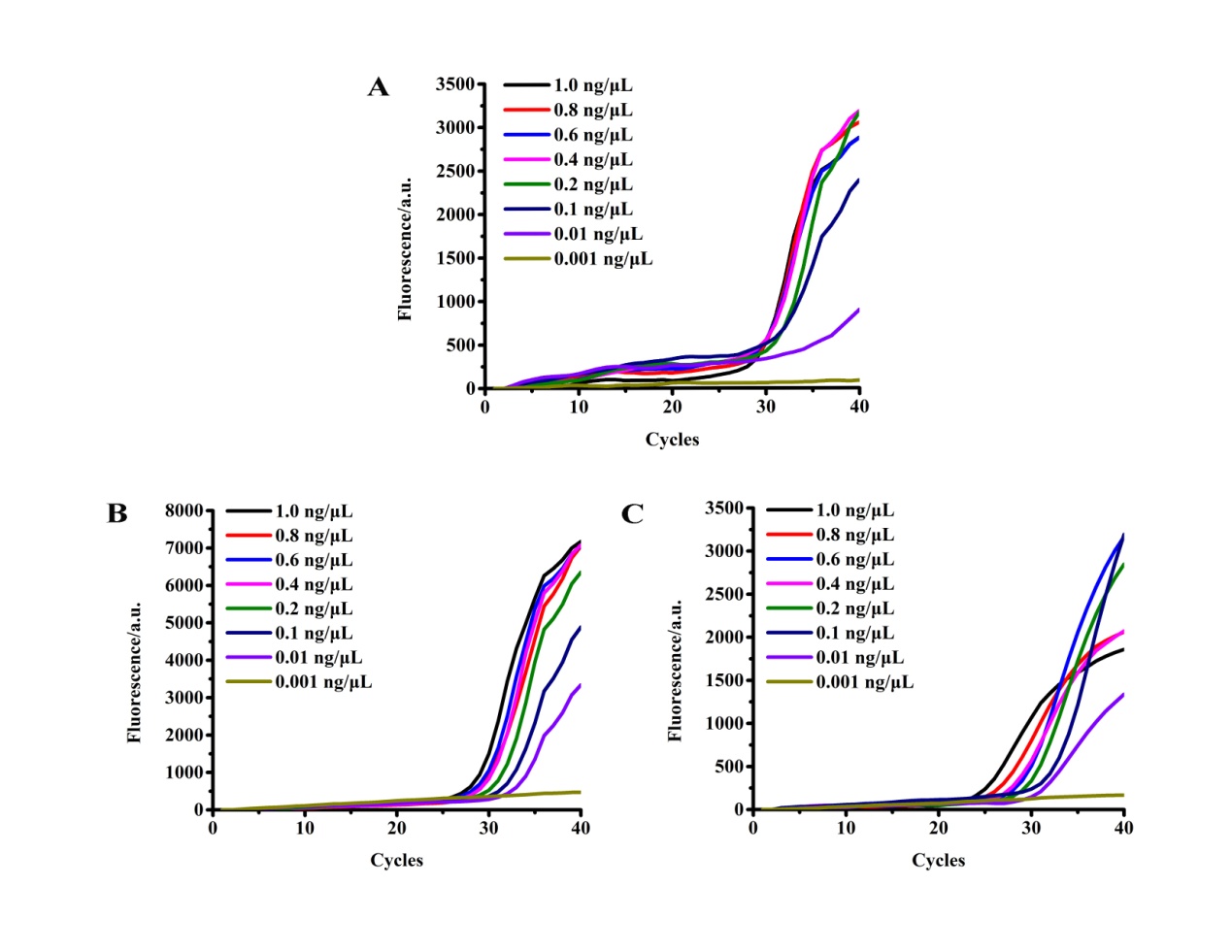


Fig. S1. The sensitivities of the real-time PCR method for peanut(A), sesame(B) and soybean(C)

Table S1. The primers and probes of RT-PCR assay

| Primer name | Primer sequence |
| --- | --- |
| 18 sRNA-F | TCTGCCCTATCAACTTTCGATGGTA |
| 18 sRNA-R | AATTTGCGCGCCTGCTGCCTTCCTT |
| 18 sRNA-Probe | FAM-CCGTTTCTCAGGCTCCCTCTCCGGAATCGAAC-TAMRA |
| Peanut-F | GCAACAGGAGCAACAGTTCAAG |
| Peanut-R | CGCTGTGGTGCCCTAAGG |
| Peanut-Probe | FAM-AGCTCAGGAACTTGCCTCAACAGTGCG-TAMRA |
| Sesame-F | CCAGAGGGCTAGGGACCTTC |
| Sesame-R | CTCGGAATTGGCATTGCTG |
| Sesame-Probe | FAM-TCGCAGGTGCAACATGCGACC-TAMRA |
| Soybean-F | GCCCTCTACTCCACCCCCA |
| Soybean-R | GCCCATCTGCAAGCCTTTTT |
| Soybean-Probe | FAM-AGCTTCGCCGCTTCCTTCAACTTCAC-TAMRA |
